# Supplementary material for: Alterations of the Mucosal Immune Response and Microbial Community of the Skin upon Viral Infection in Rainbow Trout (Oncorhynchus mykiss)
Source: Int J Mol Sci. 2022 Nov 14;23(22):14037. doi: 10.3390/ijms232214037 (PMC9698461; doi:10.3390/ijms232214037)
Supplement: Supplementary file 1 [file ijms-23-14037-s001.zip › Figure S1.pdf]

Supplementary Figure S1

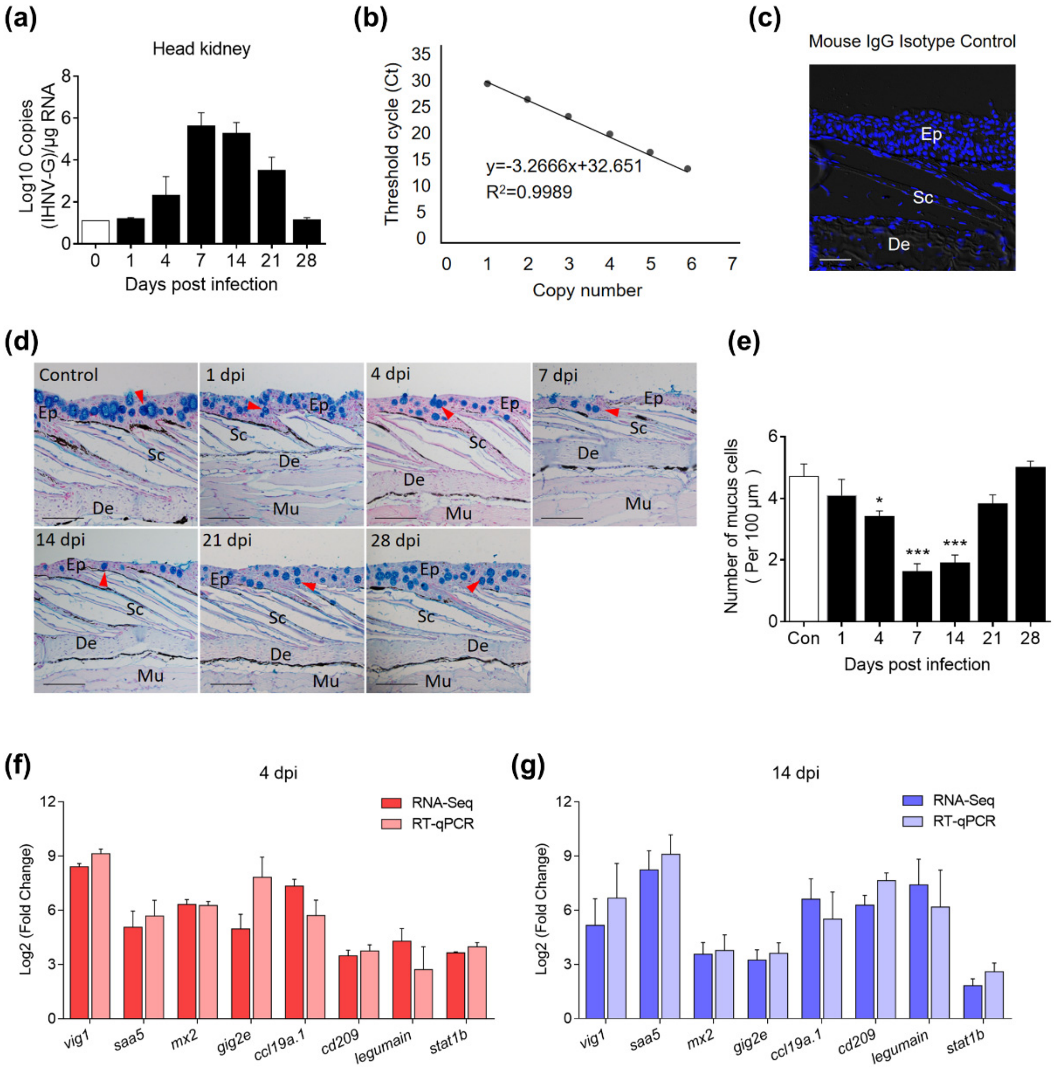

Figure S1. (a) IHNV-G gene viral load (log10) at the indicated time points after IHNV infection was assessed in head kidney samples using a RT-qPCR assay. (b) Standard curve for IHNV load used in this study. (c) Isotype control staining for anti-IHNV-N antibody in skin paraffin-sections from infected fish (7 dpi). (d) Alician blue (AB) staining of skin paraffin sections at the indicated time points (n = 6). Ep, Epidermis; Sc, Scale; De, Dermis; Scale bars, 100 µm. Red arrows indicate mucus cells within the epidermis layer of the skin. (e) Statistical histogram of the number of mucus cells in the epidermal layer of skin. Y-axis represents the average number of mucus cells per 100 µm of skin. (f-g) Validation of RNA-seq on selected genes was performed by RT-qPCR. Error bars indicate standard error.
