# Supplementary material for: Alterations of the Mucosal Immune Response and Microbial Community of the Skin upon Viral Infection in Rainbow Trout (Oncorhynchus mykiss)
Source: Int J Mol Sci. 2022 Nov 14;23(22):14037. doi: 10.3390/ijms232214037 (PMC9698461; doi:10.3390/ijms232214037)
Supplement: Supplementary file 1 [file ijms-23-14037-s001.zip › Table S1 S2 S3 S6.pdf]

Table. S1. Sequence information for primers used in this study.

Gene	GenBank accession no.	Primer Sequence (5'-> 3')
IHNV-G	NC_001652.1	F: CACGGAAACAACACCACCATTA<br>R: AACAGCAAGGAGGAGAACAAGG
EF1α	NM_001124339.1	F: CAACGATATCCGTCGTGGCA<br>R: ACAGCGAAACGACCAAGAGG
β-actin	XM_036973727.1	F: CCGCGACCTCACAGACTACC<br>R: GTGCCCATCTCCTGCTCAAA
mx1	XM_021567440.1	F: GATGCTGCACCTCAAGTCCTACTA<br>R: CGGATCACCATGGGAATCTGA
ddx58	XM_036973405.1	F: CAGAGGTACTACAGGAAATGG<br>R: TTACTGGTCTTCAAGCAATG
dhx58	XM_036940961.1	F: AGTTTGGCACGCAGGAGTA<br>R: CAAGCAGGAAGAAGTCGGT
stat1	XM_021579196.2	F: CTCATCCCCTGGACCAAGTT<br>R: TTATTGTAGCCCTCCACCCA
ifih1	NM_001195179.1	F: CAGTGGAGATGACGATGGG<br>R: ACTTGGCGTTCTTGTGCTT
tlr3	NM_001124578.1	F: GGGAATCTGACACGGCTCAA<br>R: GGAGACTCATCAAACCCGCA
c3	XM_036947707.1	F: AGAAGGTGGGAGGGGGATAC<br>R: ACTTGGTGACTGATGCCCTG
il6	CCV01624.1	F: ATTTCATCGTTCTCACAGC<br>R: ACTACCTCAGCAACCTTCA
cath1	NM_001124480.1	F: CTGGAGGCAAGCAACAAC<br>R: CCCCCAAGACGAGAGACA
vig1	NM_001124253.1	F: CAGGCCAGGTGAAGACTAGTAA<br>R: GCTGCAAAGATGCACTGGAG
saa5	XM_021586448.2	F: CGAGGACATGAGGACTCAGC<br>R: TGTTTGGATTGTGGTCCTTTCA
mx2	NM_001124751.1	F: AGGGAGTTGGAAAGGGTTACCT<br>R: CCAGACGCTGACTGGCAATA
gig2e	XM_036944105.1	F: GGAATGCAGAACCATGTGGC<br>R: TTTTCCTGTGCCTTCGCAGA
ccl19a.1	XM_036936451.1	F: ACGTCTGCTACCAGGTGACT<br>R: CCCCTAACCTGAGGCTGGTA
cd209	NM_001124633.1	F: GCCGTGTGACCACATCTACA<br>R: AACCGAGTTCAGTTCCCAGT
legumain	XM_036955355.1	F: CGGGCGATAACCACGAGAAT<br>R: GTCTACTCGGACCCCTCCTT
stat1b	XM_021579195.2	F: CGCAACTACAAGGTGATGGC<br>R: CGATGGGGCAAGACTACCAAT

Table. S2. Summary of sequencing and assembly statistics for the transcriptome data.

Sample	Raw Reads Number	Clean Reads Number	Clean Reads Rate(%)	Q20 rate(%)	Q30 rate(%)	GC content	Total mapped
Control 1	49371190	44388328	89.9	96.97	93.04	48.8	40099894 (90.34%)
Control 2	46036276	41676344	90.52	96.85	92.81	49.16	37561300 (90.13%)
Control 3	48273392	43388684	89.88	96.63	92.47	48.69	39029723 (89.95%)
4 dpi_1	52559076	47391380	90.16	96.57	92.25	48.05	42040879 (88.71%)
4 dpi_2	54021116	48408900	89.61	96.93	92.99	48.78	43713489 (90.30%)
4 dpi_3	44734772	39769710	88.9	97.07	93.21	47.53	35662480 (89.67%)
14 dpi_1	50206686	45734156	91.09	97.43	93.73	48.81	41857943 (91.52%)
14 dpi_2	50728900	45820868	90.32	96.96	92.93	48.64	41299349 (90.13%)
14 dpi_3	45721264	41230876	90.17	96.99	92.9	48.72	37243327 (90.33%)

Table. S3. The statistical results of Anosim and Permanova.

	Anosim		Permanova	
	R-value	P-value	Pseudo-F	P-value
Control-4 dpi	0.661111	0.001	6.028944	0.003
Control-14 dpi	0.747093	0.001	8.636558	0.001
4 dpi-14 dpi	0.312016	0.003	1.887767	0.014
All	0.579937	0.001	5.28605	0.001

Table. S6. Significant differences of different taxa determined by non-parametric analysis using the Kruskall-Wallis test followed by Mann-Whitney test.

**Level**	**Taxa**	**Kruskal-Wallis （p value）**	**Mann-Whitney test**	
			**4 dpi *vs* Con**	**14 dpi *vs* Con**
**Phylum**	Proteobacteria	0.0002	**	***
	Actinobacteria	0.0037	*	**
	Firmicutes	0.2594	ns	ns
	Bacteroidetes	0.1412	ns	ns
	Cyanobacteria	0.8694	ns	ns
	Verrucomicrobia	0.2201	ns	ns
	Acidobacteria	0.8470	ns	ns
	[Thermi]	0.0193	ns	ns
	Thermotogae	0.5587	ns	ns
	Fusobacteria	0.5261	ns	ns
**Order**	Actinomycetales	0.0037	*	**
	Rickettsiales	0.0001	**	***
	Vibrionales	0.0151	ns	*
	Pseudomonadales	0.1494	ns	ns
	Oceanospirillales	0.1842	ns	ns
	Lactobacillales	0.4429	ns	ns
	Burkholderiales	0.5099	ns	ns
	Flavobacteriales	0.0481	ns	ns
	Bacillales	0.5689	ns	ns
	Clostridiales	0.0430	ns	*
**Genus**	*Rhodococcus*	0.0123	*	*
	Vibrio	0.0085	ns	*
	Halomonas	0.1530	ns	ns
	Acinetobacter	0.0008	ns	*
	Streptococcus	0.9989	ns	ns
	Pseudoalteromonas	0.0579	ns	*
	Paracoccus	0.3679	ns	ns
	Corynebacterium	0.8013	ns	ns
	Salinivibrio	0.7175	ns	ns
	Flavobacterium	0.0105	ns	*
	Lactobacillus	0.2505	ns	ns
	Bacillus	0.2889	ns	ns
	Arthrobacter	0.1346	ns	ns
	Brevibacterium	0.0842	ns	ns
	Uruburuella	0.0045	ns	**
	Aeromonas	0.7487	ns	ns
	Acidovorax	0.3359	ns	ns
	Bacteroides	0.0573	ns	*
	Rubritalea	0.4835	ns	ns
	Chryseobacterium	0.0011	ns	*
